# Anti-Diabetes Drug Pioglitazone Ameliorates Synaptic Defects in AD Transgenic Mice by Inhibiting Cyclin-Dependent Kinase5 Activity

**DOI:** 10.1371/journal.pone.0123864

**Published:** 2015-04-14

**Authors:** Jinan Chen, Shenghua Li, Wenshan Sun, Junrong Li

**Affiliations:** Department of Neurology, Jiangning Hospital, Nanjing Medical University, Jiangsu, China; University of S. Florida College of Medicine, UNITED STATES

## Abstract

Cyclin-dependent kinase 5 (Cdk5) is a serine/threonine kinase that is activated by the neuron specific activators p35/p39 and plays many important roles in neuronal development. However, aberrant activation of Cdk5 is believed to be associated with the pathogenesis of several neurodegenerative diseases, including Alzheimer’s disease (AD) and Parkinson’s disease (PD). Here in the present study, enhanced Cdk5 activity was observed in mouse models of AD; whereas soluble amyloid-β oligomers (Aβ), which contribute to synaptic failures during AD pathogenesis, induced Cdk5 hyperactivation in cultured hippocampal neurons. Inhibition of Cdk5 activity by pharmacological or genetic approaches reversed dendritic spine loss caused by soluble amyloid-β oligomers (Aβ) treatment. Interestingly, we found that the anti-diabetes drug pioglitazone could inhibit Cdk5 activity by decreasing p35 protein level. More importantly, pioglitazone treatment corrected long-term potentiation (LTP) deficit caused by Aβ exposure in cultured slices and pioglitazone administration rescued impaired LTP and spatial memory in AD mouse models. Taken together, our study describes an unanticipated role of pioglitazone in alleviating AD and reveals a potential therapeutic drug for AD curing.

## Introduction

Alzheimer’s disease (AD), characterized by synaptic failures and cognitive impairment, has become a global threat to the public health [1,2]. However, the fact that there are no effective clinical drugs for AD yet suggests that it is crucial to develop some new therapeutic interventions that based on aberrant cellular and molecular signaling pathways in AD.

One of the signaling molecules that could be a potential therapeutic target is cyclin-dependent kinase 5 (Cdk5), a proline-directed serine/threonine kinase. Cdk5 is activated by the neuron specific activators, p35 or p39; and plays many important roles in neuronal migration, dendritic development and synaptic plasticity. However, deregulation, in particular, hyper-activation of Cdk5 is one key contributor to the pathogenesis of some neurodegenerative diseases including Alzheimer’s disease (AD) and Parkinson’s disease (PD)[3,4]. Indeed, it has been reported that Cdk5 activity, is robustly upregulated in postmortem AD brains[5,6]. Furthermore, suppression of Cdk5 activity pharmacologically or genetically can prevent neuronal loss and exert some protective effect in mouse models of PD[7]. Collectively, these findings suggest that inhibition of Cdk5 activity can be a promising therapeutic strategy for AD intervention.

The thiazolidinediones (TZD) drug pioglitazone is a synthetic ligand that activates the nuclear receptor peroxisome proliferator-activated receptor γ (PPARγ) and is used to treat type 2 diabetes mellitum (DM) for its insulin sensitization effect[8,9]. Previously it is reported that the TZD drugs can inhibit Cdk5-dependent hyper-phosphorylation of tau, which is an important pathological mark in AD progression[9]. Moreover, several studies also indicate the beneficial effects of TZD drugs in ameliorating some neurodegenerative diseases[10–14]. Therefore, it is of great interest to examine whether the anti-diabetes drug pioglitazone can alleviate synaptic dysfunctions and cognitive impairment in mouse models of AD by inhibiting Cdk5 kinase activity.

In this current study, we demonstrated that Aβ-induced dendritic spine loss was mediated by Cdk5 hyper-activation. Inhibition of Cdk5 either pharmacologically or genetically reversed Aβ-induced dendritic spine loss. Furthermore, we revealed that the anti-diabetes drug pioglitazone could suppress Cdk5 hyper-activation in the APP/PS1 mutant mouse hippocampus by decreasing p35 protein level. More importantly, pioglitazone can reverse LTP deficits and improve impaired spatial memory in mouse models of AD, implicating an exciting possibility that the anti-diabetes drug pioglitazone can be a promising drug for AD alleviation.

## Materials and Method

### Mice

The experimental protocol was approved by the Institutional Animal Care and Use Committee of Nanjing Medical University. We made every effort to minimize the number of mice used and their suffering. The APP/PS1 (APPSWE + PSEN1dE9) transgenic mice were purchased from the Jackson Laboratory.

### Reagents

All chemicals were purchased from Sigma unless otherwise stated. Antibodies to Cdk5 (C8), p35 (C19) were purchased from Santa Cruz. Phospho-histone H1 (32078) was purchase from Upstate. Horseradish peroxidase-conjugated goat antibodies to mouse and rabbit were from Cell Signaling.

Cdk5-shRNA(M) Lentiviral Particles (sc-35047-V) were purchased from Santa Cruz.

### Primary neuron culture and transfection of primary neuron

Hippocampal neurons were prepared from embryonic day (E) 18 rat embryos, seeded on cultured plates coated with poly-L-lysine (5 μg/ml) and maintained in Neurobasal medium (NB) supplemented with 2% B27 and 0.5 mM glutamine. To study Aβ-induced spine loss, hippocampal neurons at 12 days *in vitro* (DIV) were infected with lenti-virus encoding ctrl-shRNA/ Cdk5-shRNA together with green fluorescent protein (GFP) for 2 days and then changed back to Neurobasal medium.

### *In vitro* kinase assay

The *in vitro* kinase assay was performed as described previously[5]. The mouse hippocampi or cultured neurons were homogenized and lysed, then Cdk5/p35 protein complex was co-immunoprecipated (co-IP) using Cdk5 antibody (C8), and pulled down by protein-G agarose. The precipitate was then washed 3 times with lysis buffer and kinase assay was performed in kinase reaction buffer [25 mM Tris (pH 7.5), 10 mM MgCl_2_, and 100 mM ATP] containing 10 μg histone H1 peptide in a final volume of 50 μL at 30°C for 30 min, followed by western blotting using the phosphor-histone H1 antibody. The band intensity was quantified using the ImageJ software.

### Hippocampal slice preparation and Electrophysiology

Mouse brains of ~6 month-old mice were immediately dissected after sacrifice and soaked in artificial cerebrospinal fluid (aCSF). Brain slices at 300 μm were prepared and field excitatory postsynaptic potentials (fEPSPs) were recorded in the stratum radiatum of CA1 area. Long-term potentiation (LTP) was induced with a conditioning stimulus consisting of three theta burst trains delivered 60 seconds apart. Each theta burst train itself consisted of ten 5-Hz series of four 100-Hz pulses. fEPSP slopes were used as a measure of synaptic activity and slopes acquired during the last 10 minutes were averaged. The LTP results were analyzed by MED64 Mobius software and statistics was performed with GraphPad Prism software.

### Morris water maze (MWM)

The MWM test was performed as described previously [15], with some modifications. Briefly, for training, the male mouse (~12 month-old, 10~12 mice per group) was placed in the tank at four random points. The mouse was allowed to search for the platform for 90 s. Three training trials were given every day and the latency for each trial was recorded for 5 consecutive days. Probe trial was performed on day 6. The mouse was allowed to swim in the tank for 60 s without the platform, and performance was assessed on the basis of the time spent in the target quadrant in which the hidden platform was originally placed. The animal behavior was recorded by video camera and analyzed by Ethovision XT 7.0 (Noldus).

### Drug treatment

Roscovitine, pioglitazone and Aβ were prepared as stock solution in DMSO and diluted in medium/aCSF to its final concentration during experiment. The final DMSO concentration is ~0.01%. To examine the Cdk5 activity in cultured hippocampal neurons, half of the medium was changed 1 day before Aβ treatment, and neurons at 20 DIV were treated with DMSO/Aβ (500 nM) for 2 h. To examine dendritic spines of cultured hippocampal neurons, half of the medium was changed 1 day before drug treatment, and neurons at 20 DIV were pre-treated with DMSO/Ros (25 μM) for 2 h and then with DMSO/Aβ (500 nM) for another 48 h. To examine p35 level upon pioglitazone treatment, half of the medium was changed 1 day before drug treatment, and neurons at 20 DIV were pre-treated with DMSO/MG132 (10 μM) for 2 h and treated with pioglitazone for the indicated times and concentrations. For LTP experiment, the hippocampal slices were perfused with vehicle/pioglitazone (20 μM) for 1 h and DMSO/Aβ (500 nM) for another 2 h in oxygenated aCSF before LTP induction or ~6 month-old mice were intraperitoneally injected with pioglitazone (10 mg/kg) per day for 7 consecutive days. For MWM experiment, ~12 month-old mice were intraperitoneally injected with pioglitazone (10 mg/kg) per day for 15 consecutive days.

### Data analysis

Data are presented the mean ± SEM from at least 3 independent experiments. To quantify the spine density, images were captured with Olympus laser scanning confocal microscope with 60X objective lens and analyzed with Metamorph software. For animal experiments, data collection and analysis were performed blinded to the condition of the experiments. The significance of differences was determined by unpaired Student’s *t*-test or one-way ANOVA with Student–Newman–Keuls test. Error probabilities of *p* < 0.05 were considered as statistically significant.

## Results

### Enhanced Cdk5 activity in AD mouse models

**Fig 1:**
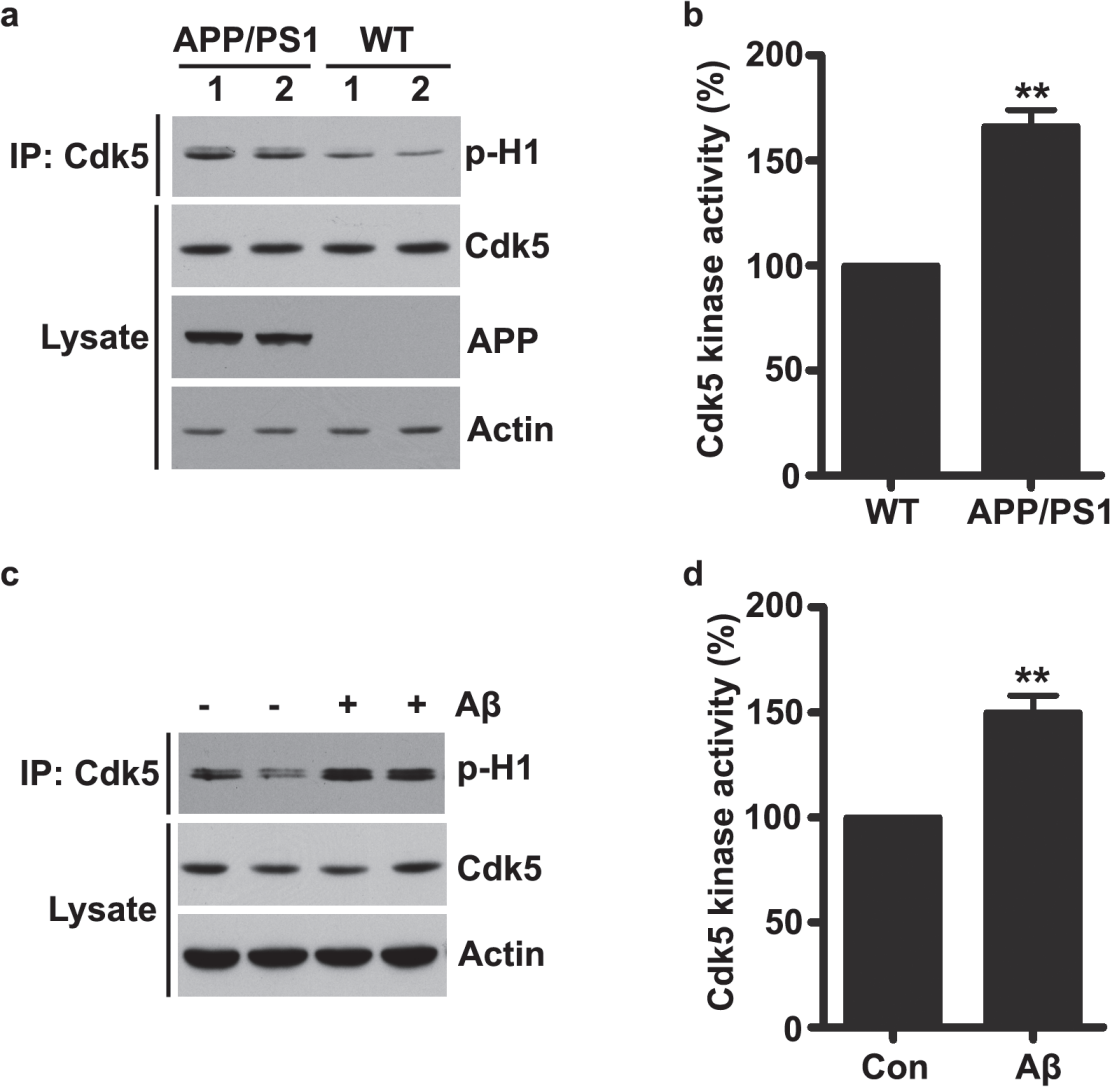
Increased Cdk5 activity in Alzheimer’s disease. (a) Hippocampi of WT and APP/PS1 mutant mice were homogenized and Cdk5 protein was immunoprecipitated and subjected to kinase assay using Histone H1 as a substrate. (b) Quantification analysis (mean ± SEM, n = 6 hippocampi; **p < 0.01, unpaired Student’s *t*-test). (c) Cultured hippocampal neurons at 20 DIV were treated with Aβ (500 nM) for 2 h and then Cdk5 protein was immunoprecipitated and subjected to *in vitro* kinase assay. (d) Quantification analysis (mean ± SEM, n = 4 independent experiments; **p < 0.01, unpaired Student’s *t*-test).

To examine whether and how Cdk5 plays a role in the synaptic dysfunctions during the pathogenesis of AD, we examined Cdk5 protein level and assessed its kinase activity by histone H1 phosphorylation via *in vitro* kinase assay in the APP/PS1 mutant mice. We found that Cdk5 activity was abnormally elevated in the 6 month-old mouse hippocampus without any obvious changes of its protein level (Fig 1A & 1B). We further examined whether the enzymatic activity of Cdk5 is affected in cultured hippocampal neurons upon Aβ treatment. Consistently, exposure to 500 nM Aβ for 2 hours significantly enhanced Cdk5 kinase activity by ~ 1.5 fold (Fig 1C & 1D). Taken together, these results suggest that Cdk5 activity was abnormally elevated in the progression of AD.

### Inhibition of Cdk5 reversed dendritic spine loss upon Aβ treatment

**Fig 2:**
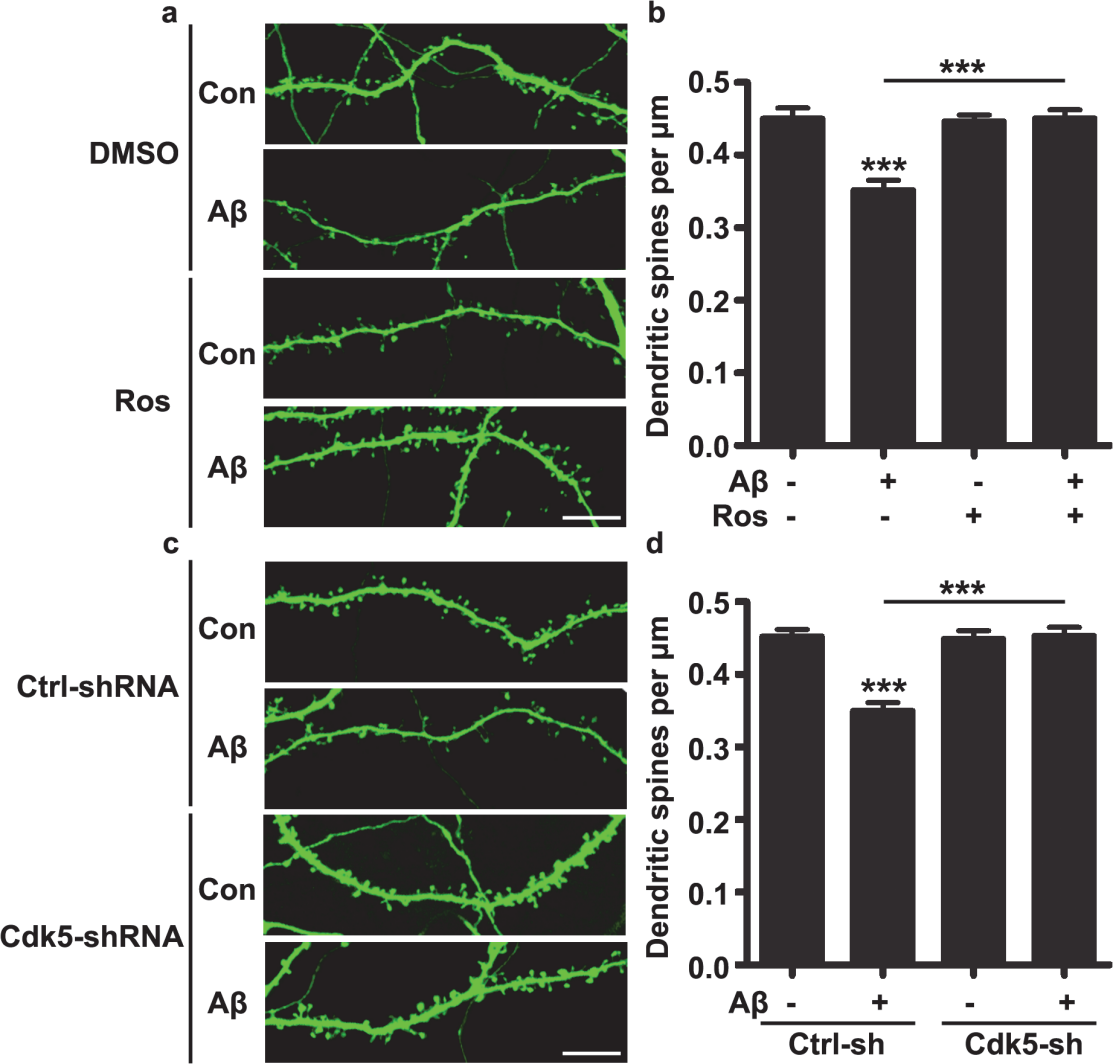
Inhibition of Cdk5 activity reversed Aβ-induced dendritic spine loss. (a) Cultured hippocampal neurons were infected with lenti-virus encoding green fluorescent protein (GFP) at 12 DIV and treated with Aβ (500 nM) for 48 hours in the presence or absence of roscovitine (25 μM) at 20 DIV. The dendritic spine density was quantified. Scale bar: 10 μm. (b) Quantification analysis (mean ± SEM, n = 30 dendrites from 10 neurons, 3 independent experiments; ***p < 0.001, one-way ANOVA with Student–Newman–Keuls test). (c) Cultured hippocampal neurons were infected with lenti-virus encoding control-shRNA or Cdk5-shRNA together with GFP at 12 DIV and treated with Aβ (500 nM) for 48 hours at 20 DIV. The dendritic spine density was quantified. Scale bar: 10 μm. (d) Quantification analysis (mean ± SEM, n = 30 dendrites from 10 neurons, 3 independent experiments; ***p < 0.001, one-way ANOVA with Student–Newman–Keuls test).

In the human AD brains and mouse models of AD, synaptic impairment represents an early pathological manifestation. It is well established that Aβ exposure led to dendritic spine loss; therefore we examined whether Aβ-induced dendritic spine loss was mediated by aberrant Cdk5 activation. Cultured hippocampal neurons were treated with Aβ in the presence or absence of the specific Cdk5 inhibitor, roscovitine. Interestingly, we found that Aβ treatment for 48 hours resulted in a significant reduction of dendritic spine density; whereas pretreatment with roscovitine completely abolished Aβ-induced dendritic spine loss (Fig 2A & 2B). Moreover, to exclude the non-specific effect of roscovitine treatment, we validated this result by silencing Cdk5 expression using lentivirus-mediated shRNA knockdown. Consistently, silencing Cdk5 expression and hence suppressing its kinase activity, restored the dendritic spine density to normal level that statistically indistinguishable to the untreated control group (Fig 2C & 2D). These results are consistent with the notion that Cdk5 hyper-activation contributes to Aβ-induced dendritic spine loss.

### Pioglitazone inhibits Cdk5 activity by decreasing p35 protein level

**Fig 3:**
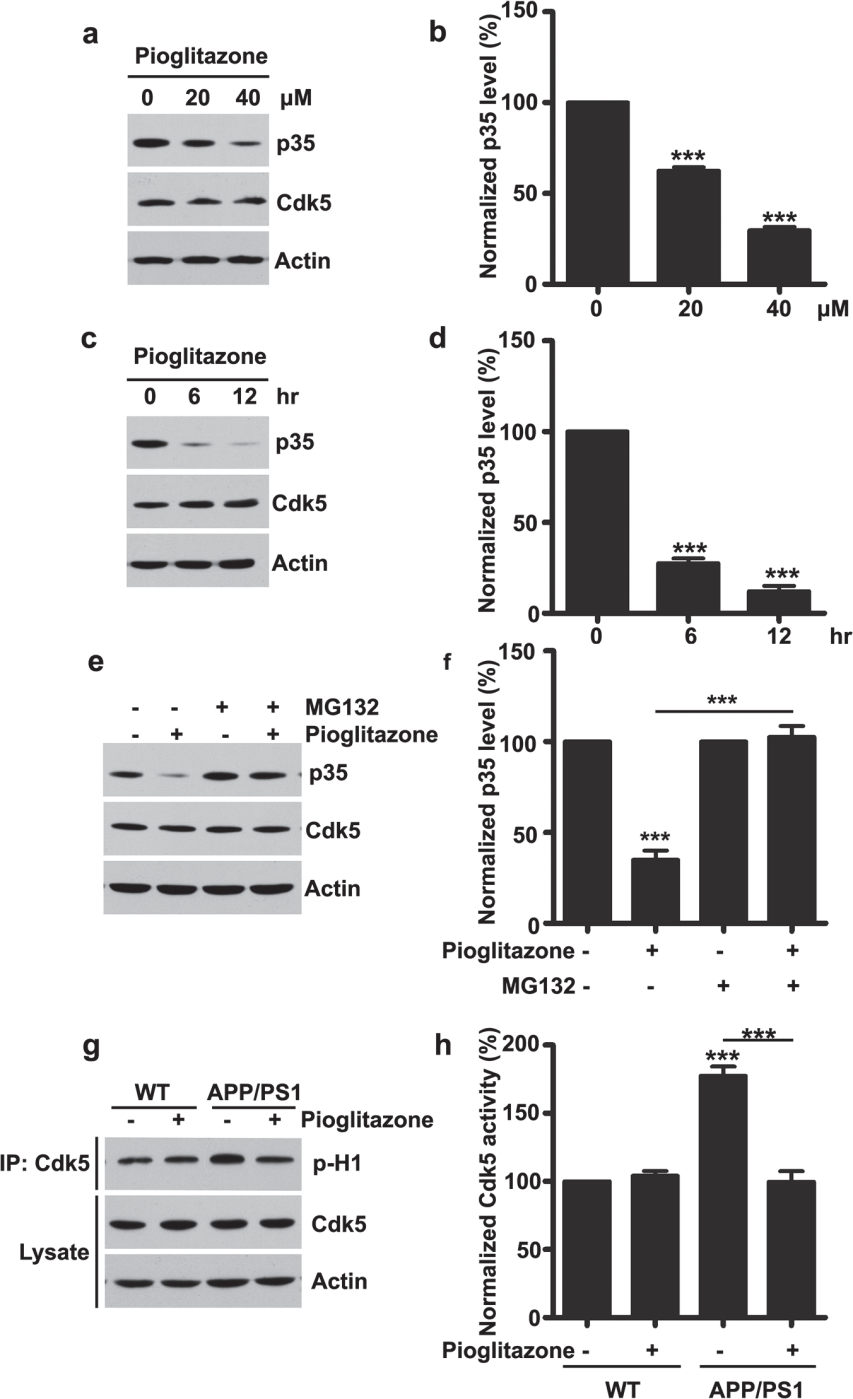
Pioglitazone inhibits Cdk5 activity by reducing p35 level. (a) Cultured hippocampal neurons at 20 DIV were treated with pioglitazone for the indicated concentration for 2 h and subjected to western blotting. (b) Quantification analysis (mean ± SEM, n = 4 independent experiments; ***p < 0.001, one-way ANOVA with Student–Newman–Keuls test). (c) Cultured hippocampal neurons at 20 DIV were treated with pioglitazone (20 μM) for the indicated times and subjected to western blotting. (d) Quantification analysis (mean ± SEM, n = 4 independent experiments; ***p < 0.001, one-way ANOVA with Student–Newman–Keuls test). (e) Cultured hippocampal neurons at 20 DIV were treated with pioglitazone (20 μM) for the 2 h in the presence or absence of MG132 (10 μM) and subjected to western blotting. (f) Quantification analysis (mean ± SEM, n = 4 independent experiments; ***p < 0.001, one-way ANOVA with Student–Newman–Keuls test). (g) WT and APP/PS1 mutant mice were treated with pioglitazone (10 mg/kg) for 7 days and then mouse hippocampi were dissected and subjected to Cdk5 kinase assay. (h) Quantification analysis (mean ± SEM, n = 4 independent experiments; ***p < 0.001, one-way ANOVA with Student–Newman–Keuls test).

Previously it is reported that the anti-diabetes drug, troglitazone/pioglitazone can inhibit Cdk5 activity and the phosphorylation of its substrate tau in both SH-SY5Y cells and primary cultured cortical neurons[9]. This raises a very interesting possibility that the anti-diabetes drug may be used to rescue synaptic deficits in AD pathogenesis by inhibiting Cdk5 kinase activity. To test this hypothesis, the effect of pioglitazone exposure was examined in cultured hippocampal neurons. Interestingly, we found that treatment of hippocampal neurons with pioglitazone decreased p35 level in a time and dose-dependent manner (Fig 3A–3D). We further confirmed that pioglitazone promoted p35 degradation in a proteasome-dependent manner. Treatment of hippocampal neurons with the proteasome inhibitor, MG132, totally blocked pioglitazone-induced p35 reduction (Fig 3E & 3F). Furthermore, we examined whether pioglitazone administration in APP/PS1 mutant mice would affect Cdk5 kinase activity. Whereas Cdk5 activity was aberrantly upregulated in APP/PS1 mouse hippocampi, pioglitazone administration in APP/PS1 mutant mice for 7 consecutive days significantly reduced Cdk5 activity to normal level (Fig 3G & 3H), confirming that the anti-diabetes drug pioglitazone can suppress Cdk5 hyper-activation in AD mouse models by reducing p35 level.

### Pioglitazone rescues LTP defects in APP/PS1 mutant mice

**Fig 4:**
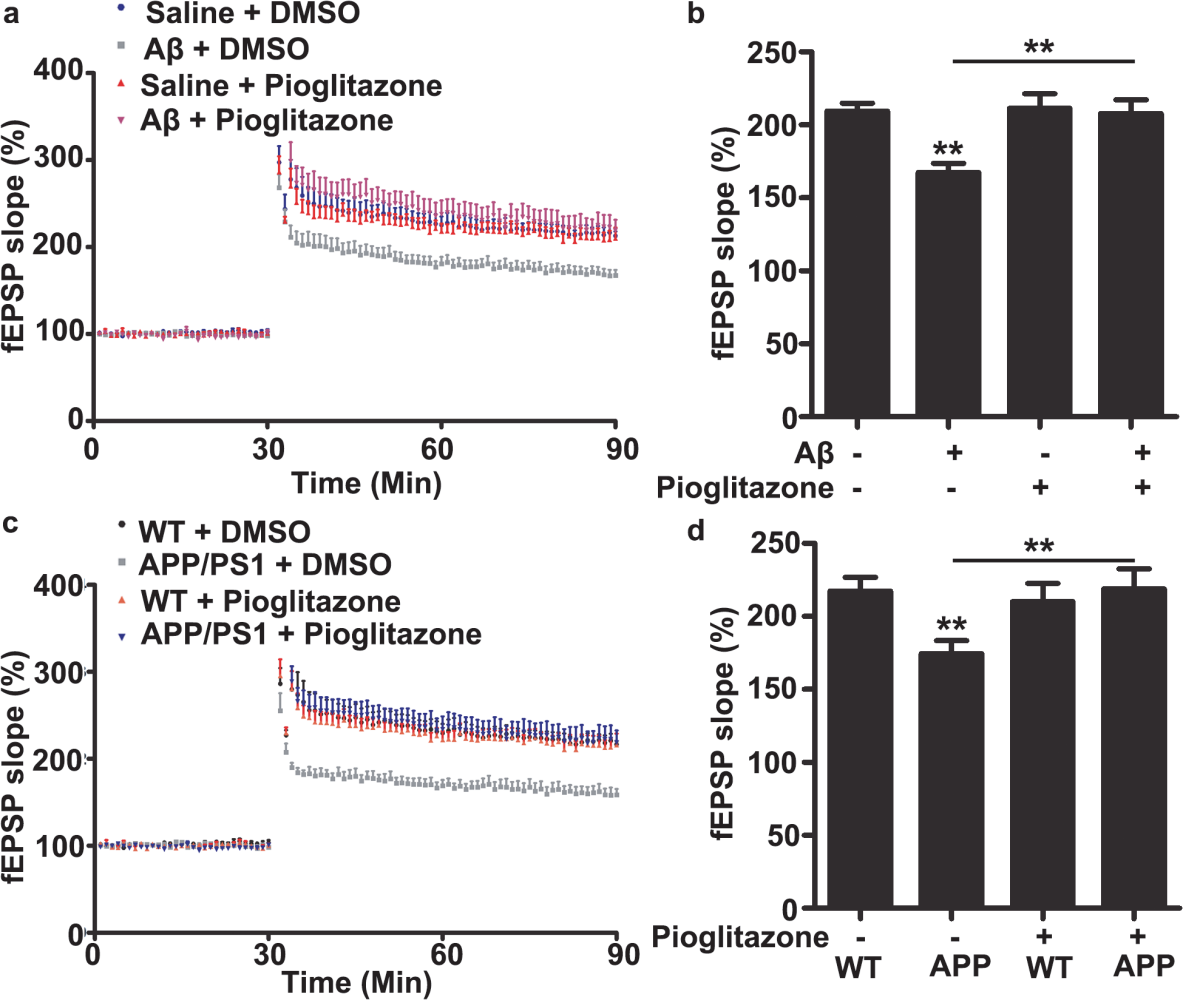
Pioglitazone rescues the CA3-CA1 LTP impairment in mouse AD models. (a) Acute hippocampal slices were treated with DMSO/Aβ (500 nM) in the presence of DMSO/pioglitazone (20 μM). (b) Quantification of the fEPSP slope 50–60 min after TBS stimulation (mean ± SEM, n = 12–16 slices from 6–8 mice; **p < 0.01, one-way ANOVA with Student–Newman–Keuls test). (c) WT and APP/PS1 mutant mice were treated with DMSO/pioglitazone (10 mg/kg) for 7 days and then LTP at CA3-CA1 synapses were recorded. (d) Quantification of the fEPSP slope 50–60 min after TBS stimulation (mean ± SEM, n = 12–16 slices from 6–8 mice; **p < 0.01, one-way ANOVA with Student–Newman–Keuls test).

Based on the observation that Cdk5 is hyper-activated and pioglitazone can inhibit its kinase activity in APP/PS1 mutant mice, it is of great interest to examine whether pioglitazone can rescue the synaptic defects in AD mouse brains. Long-term potentiation (LTP) is one important form of synaptic plasticity and is crucial for hippocampus-dependent learning and memory and it is well established that LTP impairment is one important feature in AD progression. Therefore, we examined whether pioglitazone treatment on Aβ-induced LTP defect at hippocampal CA3-CA1 synapses. Theta burst stimulation (TBS)-induced CA3-CA1 LTP was impaired in acute hippocampal slices after Aβ treatment for 2 hours, whereas co-treatment with pioglitazone abolished the Aβ-induced LTP impairment (Fig 4A & 4B). We next examined whether pioglitazone administration could rescue the impaired synaptic plasticity in APP/PS1 mutant mice. Impaired LTP was observed in ~6 month-old APP/PS1 mutant mice when compared to control littermates, more importantly, inhibition of Cdk5 hyper-activation by administration of pioglitazone for 7 consecutive days restored LTP expression (Fig 4C & 4D). Altogether, these findings show that inhibition of Cdk5 activity by pioglitazone could rescue LTP defect at CA3-CA1 synapses in APP/PS1 mutant mice.

### Pioglitazone improves spatial memory in APP/PS1 mutant mice

**Fig 5:**
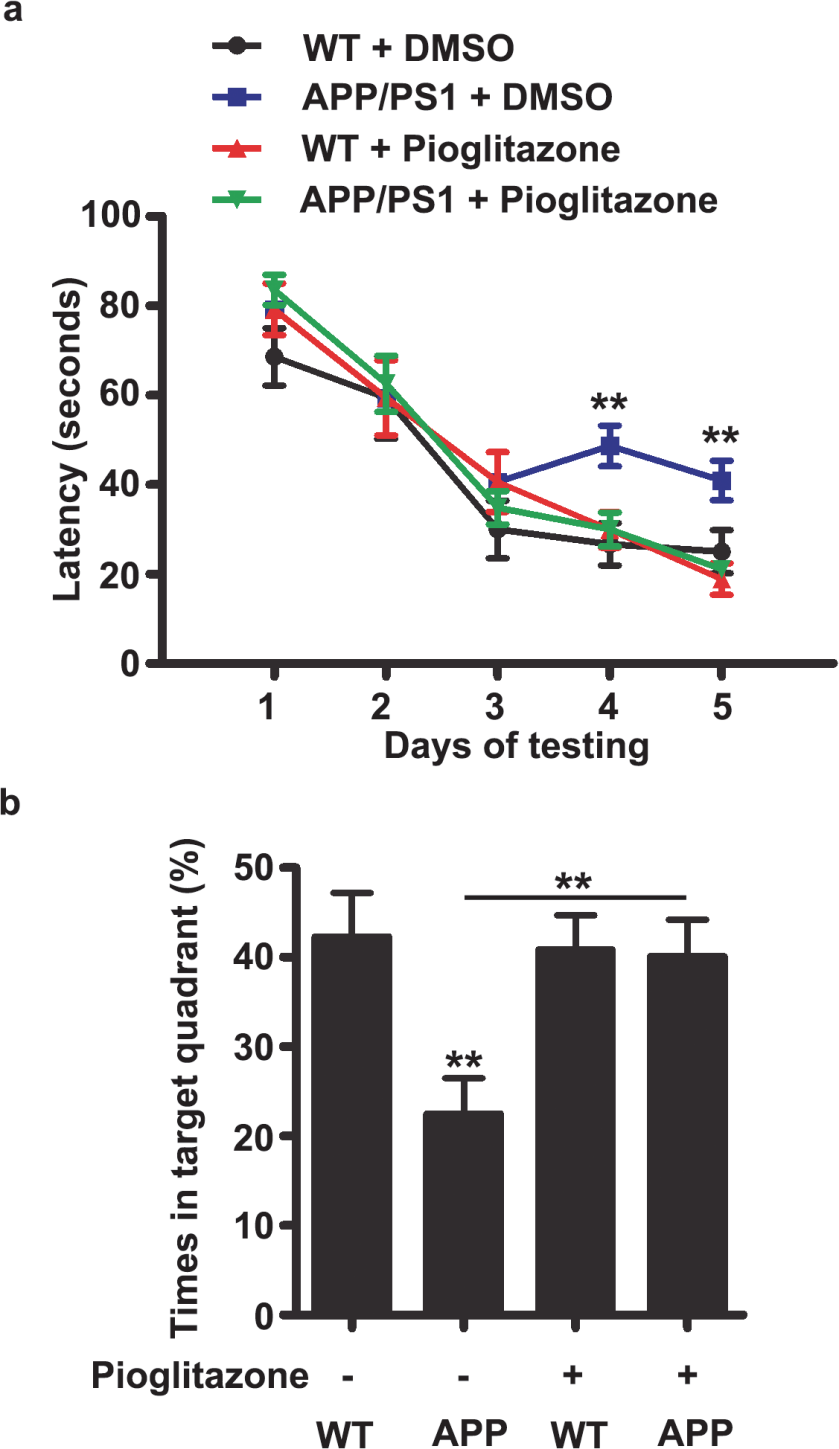
Pioglitazone improves spatial memory in APP/PS1 mutant mice. (a) WT and APP/PS1 mutant mice were treated with DMSO/pioglitazone (10 mg/kg) for 15 days and then tested in Morris water maze. Administration of APP/PS1 mice with pioglitazone significantly reduced the escape latency at D4 & 5. (b) Administration of APP/PS1 mutant mice with pioglitazone significantly enhanced the time spent in target quadrant (mean ± SEM, n = 10–12 mice; **p < 0.01, one-way ANOVA with Student–Newman–Keuls test).

Finally, we examined whether administration of pioglitazone improved hippocampus-dependent learning and memory in APP/PS1 mutant mice. We treated ~ 12 month-old APP/PS1 mutant mice and their control littermates with pioglitazone for 15 consecutive days and then examined their spatial memory using the Morris water maze (MWM) behavioral test. We trained these mice for 5 consecutive days (3 times/day) and recorded the escape latency to get to the hidden platform. We found that from Day 1 to Day 3, there were no significant differences in the escape latency among those four groups of mice, suggesting that these mice had similar motor coordination and swimming abilities. At day 4 and 5, the APP/PS1 mutant mice showed much higher escape latency, whereas pioglitazone administration significantly improved the performance of APP/PS1 mutant mice, without altering the performance of WT mice significantly (Fig 5A). After the last training day, a probe trial was performed in which the platform was removed, and the time spent in target quadrant was measured. In the probe trial, the control mice showed much more time in the target quadrant (~40%, Fig 5B), while APP/PS1 mutant mice showed no quadrant preference (~25%, Fig 5B), highlighting the spatial memory deficits of APP/PS1 mutant mice. More importantly, administration of pioglitazone in APP/PS1 mutant mice significantly improved the time spent in target quadrant (~40%, Fig 5B), suggesting that inhibition of Cdk5 activity by pioglitazone significantly improve spatial memory in APP/PS1 mutant mice.

## Discussion

A better understanding of the cellular and molecular mechanisms underlying the pathogenesis of AD can provide some promising therapeutic target for AD intervention and curing. Indeed, accumulating evidence revealed the involvement of hyper-activated Cdk5 in this devastating disease [3,16]. In the current study, we demonstrate that Cdk5 hyper-activation mediates Aβ-induced dendritic spine loss and suggest that it is a therapeutic target for AD. More interestingly, our results show that the FDA approved anti-diabetes drug pioglitazone can inhibit Cdk5 activity by decreasing p35 protein level in a proteasome dependent manner. Moreover, blockade of Cdk5 activity by pioglitazone administration can rescue impaired synaptic plasticity and spatial memory in AD mouse models, revealing an unanticipated role of pioglitazone in alleviating AD progression. These findings raise a very exciting possibility that the anti-diabetes drug pioglitazone can be used to treat AD patients in clinical trials.

Numerous studies have revealed that Cdk5 is involved in the regulation of synaptic plasticity and deregulation of its kinase activity contributes to synaptic loss and dysfunction, resulting in neuronal network impairment and cognitive decline in AD[3,16]. Nonetheless, the underlying mechanism remains unclear. Previously, it is well accepted that when neurons are exposed to cellular stress including neurotoxicity and oxidative stress, the Cdk5 activator p35 is cleaved to p25 by calpain, resulting in Cdk5 hyper-activation and its subcellular mis-localization [16,17]. However, in the present study, we did not observe any obvious p25 production in Aβ-treated neurons or APP/PS1 mouse hippocampi. Another interesting possibility is post-translational modifications of Cdk5. For example, Cdk5 S-nitrosylation, which involves a reversible attachment of nitric oxide (NO) to free cysteine residues, is implicated in Aβ-triggered spine loss and is elevated in postmortem brains of AD patients[5]. In parallel, phosphorylation of Cdk5 at tyrosine15 (Y15) significantly increased its kinase activity and resulted in dendritic spine retraction[18]. Therefore, it is interesting to examine whether Cdk5 is S-nitrosylated or phosphorylated at Y15 upon Aβ treatment or in AD mouse hippocampi. The aberrant Cdk5 activity can result in dendritic spine loss and induce cell death through the phosphorylation of different substrates. For example, the Cdk5 substrate, Wave1 is important for dendritic spine development. However, Cdk5-dependent phosphorylation of Wave1 can inhibit its activity and interrupt actin dynamics, resulting in reduced mature spines and compromised synaptic functions [19,20]. In parallel, Cdk5 hyper-activation can lead to tau hyper-phosphorylation and disrupt microtubule dynamics, triggering neuron cell apoptosis [21,22]. Therefore, the Cdk5 inhibitor, roscovitine, or some small peptides that can interrupt Cdk5/p35 interaction are under development for some neurodegenerative diseases intervention[23–25]. However, many candidates failed in the clinical trial. Here, our exciting results suggest that the FDA approved anti-diabetes drug pioglitazone can be a promising drug to prevent AD progression by decreasing p35 protein level and suppressing Cdk5 activity. However, it remains unknown how pioglitazone can effectively promotes p35 degradation. In this regard, it will be of great interest to examine the underlying mechanism by which pioglitazone controls p35 protein abundance.

It is noteworthy that the anti-diabetes drug pioglitazone have been prescribed for the treatment of type 2 diabetes mellitum (DM) in a PPARγ-dependent and-independent manner[26]. It is well known that pancreatic cells and neurons share many common features including some important regulatory cellular and molecular mechanisms. Indeed, previously it is reported that Cdk5 hyper-activation is also involved in the pathogenesis of diabetes[27–29]. In addition, some clinical studies showed that diabetes increased AD risk by 2–3 fold[30] and over 80% of AD patients showed abnormal blood glucose level and developed diabetes[31,32], indicating that the anti-diabetes drugs may also be used to treat AD. Consistently, here we found that the FDA approved anti-diabetes drug pioglitazone can inhibit Cdk5 activity in both hippocampal neurons and APP/PS1 mutant mouse hippocampi. More importantly, pioglitazone reverses the synaptic dysfunctions and improves spatial memory in AD mouse models. These findings collectively raise a very promising possibility that the anti-diabetes drug pioglitazone can be used to cure AD patients.
